# Metagenomic Sequencing of the Chronic Obstructive Pulmonary Disease Upper Bronchial Tract Microbiome Reveals Functional Changes Associated with Disease Severity

**DOI:** 10.1371/journal.pone.0149095

**Published:** 2016-02-12

**Authors:** Simon J. S. Cameron, Keir E. Lewis, Sharon A. Huws, Wanchang Lin, Matthew J. Hegarty, Paul D. Lewis, Luis A. J. Mur, Justin A. Pachebat

**Affiliations:** 1 Institute of Biological, Environmental and Rural Sciences, Edward Llywd Building, Penglais Campus, Aberystwyth, Ceredigion, SY23 3FG, United Kingdom; 2 Department of Respiratory Medicine, Prince Phillip Hospital, Hywel Dda University Health Board, Llanelli, SA14 8QF, United Kingdom; 3 College of Medicine, Swansea University, Swansea, SA2 8PP, United Kingdom; University of Tübingen, GERMANY

## Abstract

Chronic Obstructive Pulmonary Disease (COPD) is a major source of mortality and morbidity worldwide. The microbiome associated with this disease may be an important component of the disease, though studies to date have been based on sequencing of the 16S rRNA gene, and have revealed unequivocal results. Here, we employed metagenomic sequencing of the upper bronchial tract (UBT) microbiome to allow for greater elucidation of its taxonomic composition, and revealing functional changes associated with the disease. The bacterial metagenomes within sputum samples from eight COPD patients and ten ‘healthy’ smokers (Controls) were sequenced, and suggested significant changes in the abundance of bacterial species, particularly within the *Streptococcus* genus. The functional capacity of the COPD UBT microbiome indicated an increased capacity for bacterial growth, which could be an important feature in bacterial-associated acute exacerbations. Regression analyses correlated COPD severity (FEV_1_% of predicted) with differences in the abundance of *Streptococcus pneumoniae* and functional classifications related to a reduced capacity for bacterial sialic acid metabolism. This study suggests that the COPD UBT microbiome could be used in patient risk stratification and in identifying novel monitoring and treatment methods, but study of a longitudinal cohort will be required to unequivocally relate these features of the microbiome with COPD severity.

## Introduction

Chronic obstructive pulmonary disease (COPD) is a leading cause of death and morbidity, leading to an estimated 2.75 million deaths worldwide in 2006 [1]. COPD is usually caused by smoking in the developed world and is an umbrella term for a multisystemic inflammatory state, including several diseases such as chronic bronchitis and emphysema. Patients experience acute exacerbations which may be triggered by environmental pollutants or infection. Approximately 75% of acute exacerbations are attributed to viral or bacterial infection, or a combination of both [2]. Characterisation of bacteria cultured from the airways of COPD patients has linked exacerbations with pathogens such as *Streptococcus pneumoniae*, *Haemophilus influenzae* and *Mora*x*ella catarrhalis* [3]. Since the advent of culture-independent techniques, especially amplification and sequencing of the 16S rRNA gene, the lung microbiome of COPD has been more widely studied [4–10]. However, to date, these studies have had conflicting results in terms of the taxonomic composition of the lung microbiome in COPD.

Lung microbiome analyses based on 16S rRNA amplicon sequencing have compared bronchial alveolar lavages (BAL) from patients with COPD to healthy individuals. Initial studies suggested that the lung microbiome of patients with moderate and severe COPD patients is less diverse than ‘healthy’ controls [4], although other work suggested this was an underestimation of bacterial diversity [10]. More recent work, with a larger cohort of moderate and severe COPD patients, suggested increased microbial diversity in more severe COPD [5]. Due to the heterogeneous nature of COPD, the concept of a ‘core’ microbiome in the lung has proved difficult to establish for the disease. Candidate genera constituting the core microbiome, from a number of COPD lung microbiome studies, include *Pseudomonas*, *Streptococcus*, *Prevotella* and *Fusobacteria* [2].

The introduction of amplicon sequencing has allowed for a much deeper insight into the human microbiome, and its relationship with health and disease. However, because it is limited to the sequencing of a single region of DNA, it is not able to robustly describe the functional capacity of the microbiome. It has been suggested that changes in the functional capacity of the human microbiome may be of higher importance in health and disease, than changes in its taxonomic composition [11]. Shotgun metagenomics approaches allows for sequencing of the entire genomic component of the human microbiome. This means that both the taxonomic composition and functional capacity of the microbiome can be investigated in much greater detail than previously possible [12]. To the best of the authors’ knowledge, no metagenomic study of the COPD microbiome exists within the literature. Other respiratory conditions have been studied with this method, such as cystic fibrosis, though with relatively small sample numbers, such as two [13], five [14], and ten [15].

The enclosed nature of the lungs presents difficulties in terms of sampling its microbiome. A number of possible sampling biofluids are possible, such as BAL, tissue biopsy, or sputum. In this study, we have chosen to use spontaneously produced sputum as it offers a non-invasive sampling method, and thus could offer the easiest method of sample collection if analysis of the microbiome in COPD patients becomes clinically useful. Recent work has suggested that sputum and BAL samples offer spatially distinct representations of the lung microbiome. BAL samples appear to represent the lower bronchial mucosal flora and sputum samples the upper bronchial tract [8]. Therefore, care should be taken in the interpretation of COPD microbiome studies in view of the difference between spatially distinct regions of the lung.

Here we report on the direct metagenomic sequencing of sputum samples from eight COPD patients and ten ‘healthy’ smokers. Metagenomic sequencing allowed for species-level resolution and the functional properties of the microbiome to be profiled. Thus, this study pursued three main objectives, namely whether there are species-level characteristics of the COPD UBT microbiome, whether the functional capacity of the UBT microbiome is altered in COPD and lastly whether progression of COPD could be correlated with taxonomic and functional aspects of the UBT microbiome.

## Materials

### Ethics Statement and Role of Funding Source

The MedLung observational study (UKCRN ID 4682) received loco-regional ethical approval from the Hywel Dda Health Board (05/WMW01/75). All procedures undertaken within this study were in accordance with the ethical standards of the Helsinki Declaration (1964 and amended 2008) of the World Medical Association. Written informed consent was obtained from all participants at least 24 hours before sampling, at a previous clinical appointment, and all data was link anonymised before analysis. The sponsor was Hywel Dda University Health Board and neither the funders—Aberystwyth University or NISCHR—nor sponsor had any input into the design or reporting of the study. All methods were carried out in accordance with relevant guidelines and regulations.

### Patient Recruitment and Sampling

Spontaneous sputum was collected from eight patients (five male: three female) with a clinical and spirometric diagnosis of COPD from two UK hospitals (each at least ten pack year smokers (mean = 46), older than 40 years (mean = 68), and post bronchodilator FEV_1_/FVC <0.70). A definitive clinical diagnosis requires spirometry assessment, as performed with participants in this study. A post-bronchodilator forced expiratory volume in one second (FEV_1_) over forced vital capacity (FVC) ratio, also defined as FEV_1_% of predicted, the proportion of a patient’s lung capacity that they are able to expel within one second (FEV_1_) as a proportion of the total air that can be expelled from the lungs after full inspiration (FVC) of less than 0.70 confirms the presence of COPD. Of the eight COPD patients, three were classified as GOLD stage II and five as GOLD stage III. Ten (six male: four female) spontaneous sputum samples were collected from staff members, (mean age = 53) at Swansea University who were either current or ex-smokers but had no known lung disease and no symptoms of COPD (cough, chronic sputum, breathless, wheeze or chest pain). This group were treated as ‘healthy’ smokers and subsequently referred to as Control samples/participants. All spontaneous sputum samples contained bronchial cells as confirmed by a Consultant Pathologist. Further patient details are supplied in S1 Table.

### Isolation of Genomic DNA

After transferal on dry ice, sputum samples were thawed on ice for 60 minutes and then treated with 5 mL of 30% aqueous methanol and 500 μL of a methanol-dithiothreitol (DTT) solution, made up by adding 2.5 g DTT to 31 mL of 30% aqueous methanol, and then vortex mixed for 15 minutes. Samples were then underwent centrifugation at 1500 x g for ten minutes, the supernatant removed and the pellet transferred to a PCR grade 1.5 mL microcentrifuge tube. Genomic DNA was extracted from 100 μL of treated sputum using a FastDNA SPIN kit for soil (MP Biomedical, Santa Ana, USA) following manufacturer’s instructions. Bead beating was carried out in a FastPrep-24 machine (MP Biomedical) with three cycles at speed setting 6.0 for seconds, with cooling on ice for 60 seconds between cycles. Genomic DNA was eluted with 30 μL of DES and dsDNA concentration determined using the Quant-iT dsDNA High Sensitivity assay kit and a Qubit fluorometer (Life Technologies, Paisley, UK).

### Metagenomic Library Preparation and Sequencing

Extracted genomic DNA was normalised to 10 ng/μL with PCR grade water (Roche Diagnostics Limited, West Sussex, UK) and 50 ng used to create metagenomic libraries using the Nextera^®^ DNA kit (Invitrogen, San Diego, USA) following standard instructions, except that a MinElute PCR purification kit (Qiagen, Ltd Crawley, UK) was used for the clean-up of tagmented DNA. Nextera^®^ DNA libraries were quantified as above, and approximate library sizes determined by running on a 2% agarose gel alongside HyperLadder IV (Bioline, London, UK). Sample libraries were pooled in equimolar concentrations following Illumina guidelines and sequenced at 2 x 151 bp using an Illumina HiSeq 2500 rapid run, with samples duplicated over two lanes, and following standard manufacturer’s instructions at the IBERS Aberystwyth Translational Genomics Facility.

### Metagenomic Sequence Analysis

After sequencing, output files for each sample were combined into one file using the BioLinux 7 environment [16] for each read direction. Sequencing files were uploaded to MG-RAST (v3.2) [17] as FASTQ files and paired-end reads joined using the facility available within MG-RAST, with non-overlapping reads retained. Sequences were dereplicated and dynamically trimmed using the default parameters for FASTQ files and human sequences removed by screening against the *Homo sapiens* (v36) genome. The MG-RAST pipeline used an automated BLASTX annotation of metagenomic sequencing reads against the SEED non-redundant database [18]. The SEED hits can be matched to identity at various taxonomic levels; e.g. genus or species levels. Organism abundances were modelled and exported from MG-RAST using the ‘Best Hit Classification’ after alignment to the M5NR database, with only alignments with a maximum e-value of 1 x 10^−5^, minimum identity cut-off of 97%, and a minimum alignment cut-off of 15 being used. Functional abundances were modelled and exported from MG-RAST using ‘Hierarchical Classification’. SEED matches can also be related to metabolic information, again at different levels of classification. The coarsest level of organization; the generalized cellular function was termed level 1, and the finest, individual subsystems level 3. Eukaryotic taxonomic classifications were trimmed based on literature searches to remove poorly classified reads.

### Data Deposition

Sequence files can be viewed on MG-RAST via the individual sample IDs listed in S1 Table. Raw sequence reads have been deposited at the European Nucleotide Archive under primary project accession number PRJEB9034 and secondary accession number ERP010088. In line with the European Nucleotide Archive’s guidelines, host sequence reads have been removed.

### Data and Statistical Analysis

Read abundances were transformed, to normalise for potential variations in sequencing efficacy, into percentage abundance based upon the total number of sequences within each individual sample. This was completed at each taxonomic level of classification, namely genera and species, and functional level of classification, namely Level 1, Level 2, and Level 3. These normalised percentage abundance values were used in all subsequent data and statistical analyses. Principal coordinate analysis (PCA) was completed using the MG-RAST analysis pipeline using taxonomic and functional assignments, respectively, to the M5NR database with only alignments with a maximum e-value of 1 x 10^−5^, minimum identity cut-off of 97%, and a minimum alignment cut-off of 15 being used. Evaluation of significant changes between COPD and Control samples in regards to different levels of taxonomic and functional assignments was completed using the MetaboAnalyst 2.0 [19] *t*-test facility with a significance *P* value threshold of less than 0.05. The MINITAB 14 package was used for regression analyses of taxonomic and functional assignments associated with COPD severity. The MINITAB 14 package uses the computational routine Givens transformations using LINPACK routines [20]. The regression model uses the equation y = β_0_ + β_1_X_1_ + e where y equals the response, β_K_ equals the population regression coefficients, X equals the predictors, and e equals the error term with a normal distribution, mean of 0, and standard deviation of α. Within this regression model, the FEV_1_% of predicted was considered the independent variable. The pairwise correlation of variables are performed by multiple Pearson analyses using the well-established correlograms (corrgrams) programme [21]. The outputs are hierarchically clustered based on dissimilarity measures. The outputs are given in piecharts where the filled portion of the pie indicates the magnitude of the correlation and the depth of the shading indicates the magnitude of the correlation. Also provided as supplementary data are scatterplots of the correlated variables and the statistically significance of the correlations, where font size also indicated the strength of the correlations.

## Results

The group characteristics of the patient from which sputum samples were obtained were similar in terms of smoking status, age and gender (S1 Table). Following DNA isolation from samples and sequencing, one-way ANOVA indicated no statistically significant differences in average sequence number or bp number (S2 Table). Average read lengths however, were significantly longer (*P* = 0.001) in control samples, by approximately 4 bp. An average of 12.7 million sequences with an average read length of 136 bp was achieved after MG-RAST quality control processes for each sample.

**Fig 1:**
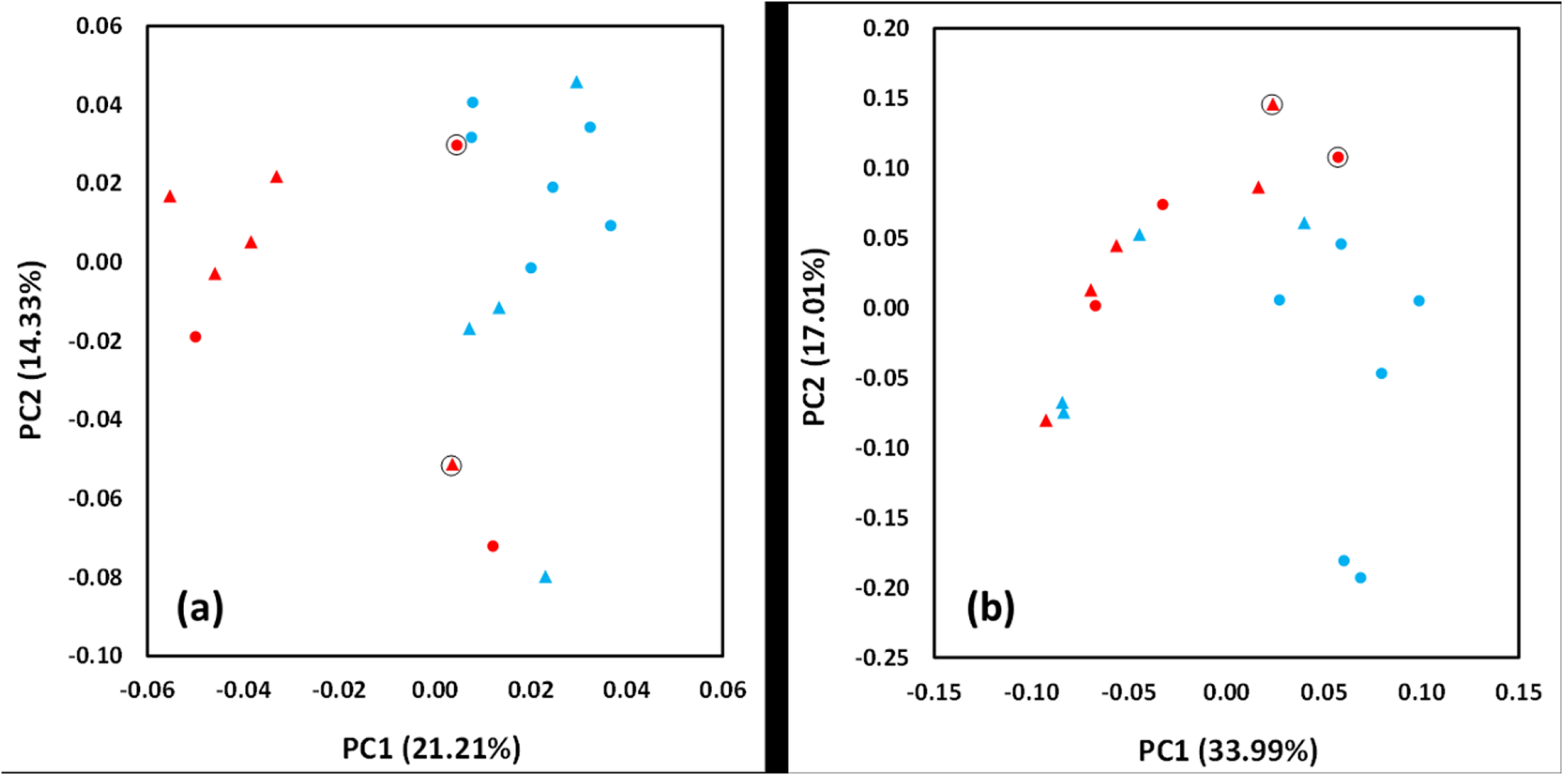
Principal component analysis of taxonomy and functional classifications. PCA plots were created using (A) taxonomic and (B) functional classifications, using the analysis method detailed previously. Control samples are coloured blue and COPD red. Triangles indicate patients who are current smokers, and black circles indicate the patient has antibiotic use in their medical history prior to giving a sample. PCA plots drawn using normalised values and Manhattan distance.

PCA revealed some separation between the control and COPD groups when considering taxonomic classification with five out of the eight COPD samples forming a distinctive cluster (Fig 1A). Assessments based on functional classification (Fig 1B) appeared to reduce the separation of the two groups but seven of the control samples clustered away from the COPD samples. In neither PCA was separation influenced by smoking status or reported prior use of antibiotics.

**Fig 2:**
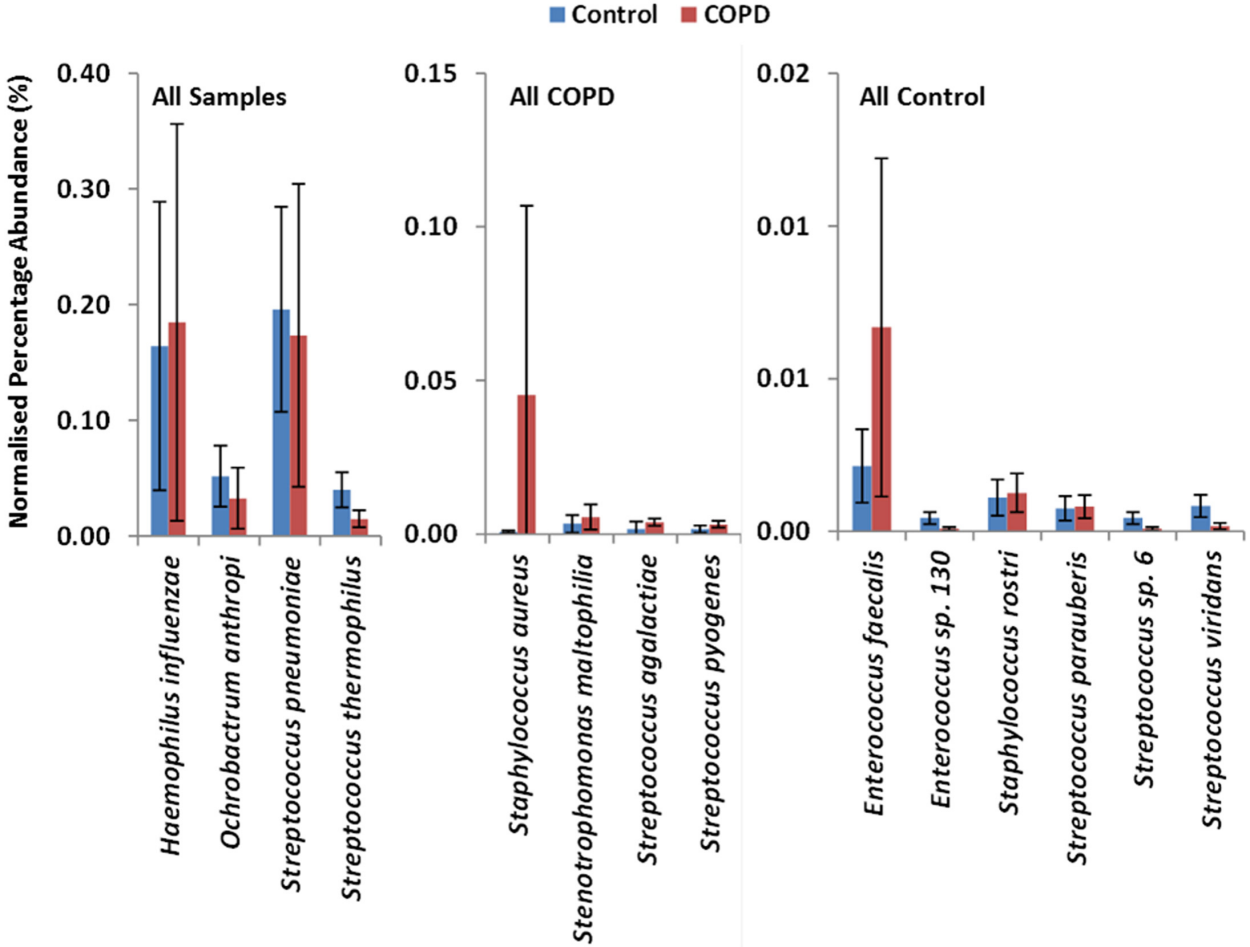
‘Core microbiome’ differences between Control and COPD participants. Abundance of the 14 bacterial species that constitute the ‘core microbiome’ in Control participants and COPD patients. Four bacterial species were found in all samples from both groups, four species were found in all of the COPD samples but not all of the Control samples, and six species were found in all of the Control samples but not all of the COPD samples. There was no bacterial species that was common to all samples in one of the two groups, but unique to that group.

We found eight bacterial genera were present in all 18 sputum samples, *Haemophilus*, *Lactobacillus*, *Neisseria*, *Ochrobactrum*, *Pseudomonas*, *Staphylococcus*, *Streptococcus*, and *Veillonella*. Five genera were found in all control samples, *Actinomyces*, *Enterococcus*, *Fusobacterium*, *Gemella*, and *Rhodococcus*, but not all COPD samples. Additionally, three genera, were found in all COPD samples, Brucella, *Stenotrophomonas*, and *Xanthomonas*, but not all control samples. Moving to consider the metagenomics outputs at the bacterial species level (Fig 2), there are four present in all 18 samples, *H*. *influenzae*, *O*. *anthropic*, *S*. *pneumoniae*, and *S*. *thermophilus*. Crucially, four additional species found in all of the COPD samples but not all control samples–*S*. *aureus*, *Stenotrophomonas maltophilia*, *Streptococcus agalactiae*, and *S*. *pyogenes*. Conversely, six species found in all control samples but not in COPD samples; namely two *Enterococcus* species, *S*. *rostri* and the *Streptococcus* species *S*. *parauaberis*, *S*. *virdans* and sp.6.

**Fig 3:**
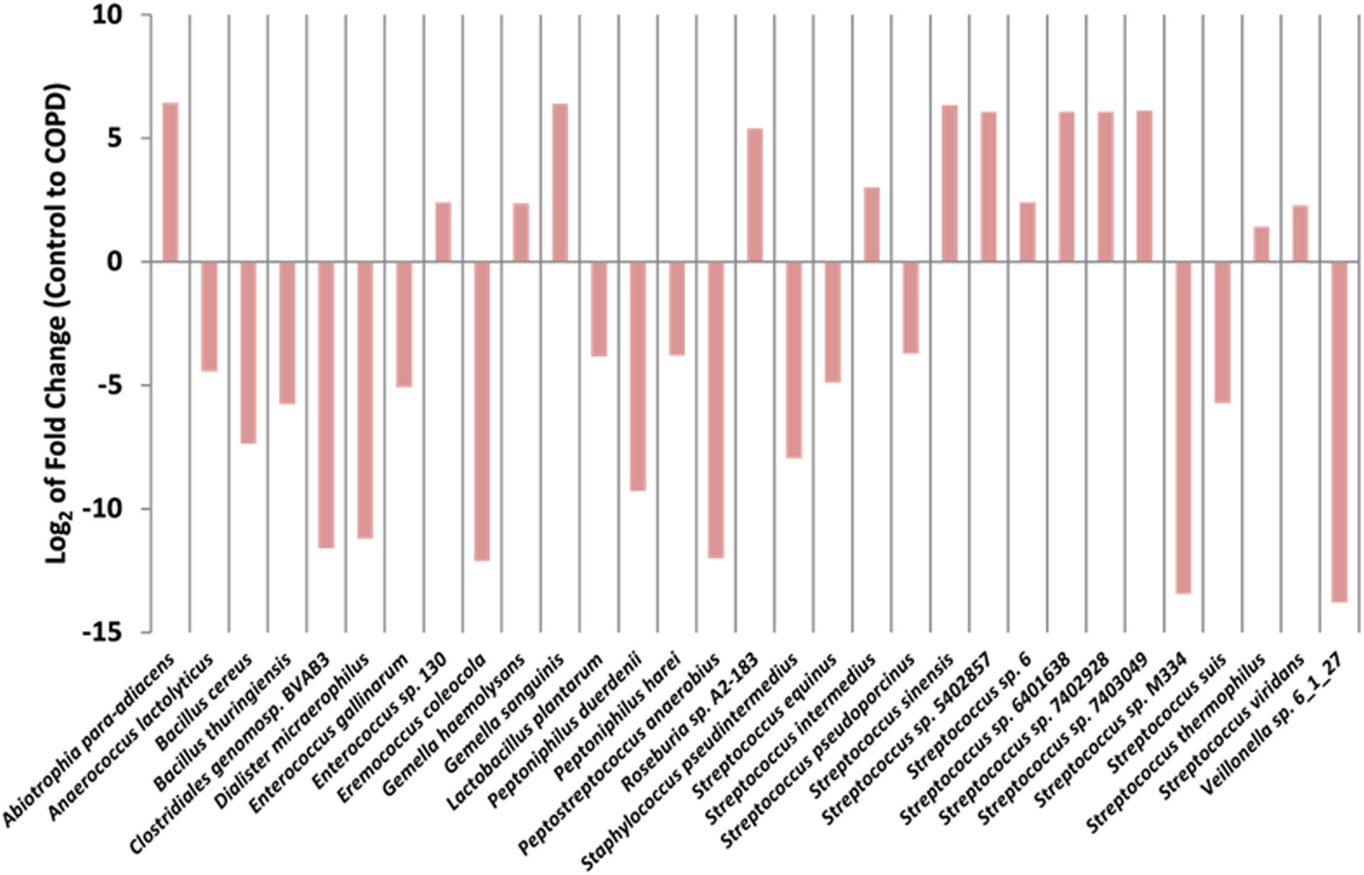
Significant changes in species abundance from Control to COPD. Using MetaboAnalyst 2.0, *t*-tests and fold-differences were calculated from normalised percentages of reads, with only those with a *P* value of < 0.05 charted. Significant differences in species abundances show both higher and lower levels in COPD samples, compared to Controls. Analysis shows that the *Streptococcus* genus is particularly dynamic.

Other species were present in all samples but exhibited statistically significant differences in fold abundance between COPD and controls (Fig 3). These species included higher abundances of the pathogens *Gemella haemolyses*, *Abiotrophia para-adiacens* and *Glemella sanginis*. Individual species within the *Streptococcus* genus in particular appeared to exhibit variable differences in abundance in COPD patient samples with both higher and lower abundances compared to the Control group observable. Non-human eukaryotic sequences were identified in the libraries but no significant differences in species abundance were detected when comparing control and COPD groups (data not shown).

**Fig 4:**
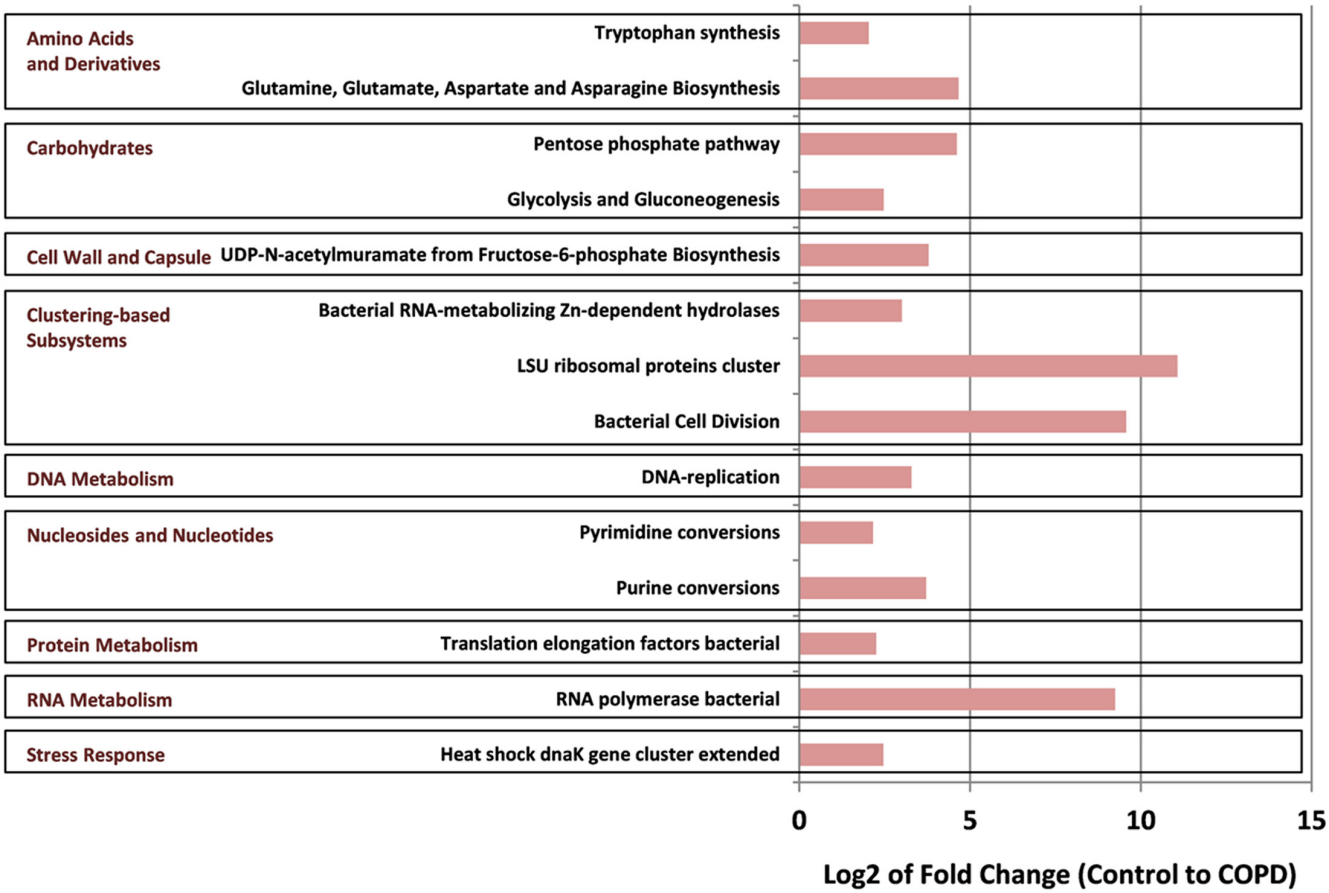
Significant differences in functional classification abundance from Control to COPD. Using MetaboAnalyst 2.0, t-Tests and fold-changes were calculated from normalised percentages of reads, with only those with a *P* value of < 0.05 charted. Functional classifications are grouped by their Level 1 classification. Only those differences at the Level 3 function are charted, with Levels 1 and 2 shown in S2 and S3 Figs respectively. Differences at Level 3 appear to centre on differences to those reads aligned to functional roles in bacterial cell division.

Considering classification categories based on bacterial gene functions significant differences in COPD versus Control sample microbiomes were detected. At the crudest functional classification; Level 1, (S1 Fig), there were significantly fewer alignments to carbohydrate genes in COPD patients but increases in clustering-based subsystems, horizontal gene transfer and nucleosides and nucleotides. At the more resolved Level 2 functional classification (S2 Fig) 26 classifications exhibited significant differences, with 22 higher in COPD patients. Only significantly higher abundance differences in COPD patients were observed at the most resolved Level 3 (Fig 4). These latter metagenomic alignments appear to centre on functional classifications involved in bacterial growth, including bacterial cell division, nucleosides and nucleotides and amino acid, protein and RNA metabolism.

In assessing the potential influence of our finding on the severity of airflow obstruction (FEV_1_% of predicted) (Table 1) we found a positive correlation with the *Streptococcus* genus (R^2^ = 51.8%, *P* = 0.044), and more specifically *S*. *pneumoniae* (R^2^ = 63.6%, *P* = 0.018). Additionally, functional positive correlations were observed with the level 2 classification of di- and oligosaccharides (R^2^ = 50.8%, *P* = 0.047) and more specifically at Level 3 with sialic acid metabolism (R^2^ = 51.1%, *P* = 0.046). We found no significant correlation between *S*. *pneumonia* and smoking pack years or age but the genus *Neisseria* showed a correlation with smoking pack years (R^2^ = 66.1%, *P* = 0.014). Notable positive functional correlations for smoking pack years were with bacterial DNA repair, potassium homeostasis and the protease modulator YbbK [22]. With regards to age, the *Ochrobactrum* genus showed a significant positive relationship with age (R^2^ = 51.6%, *P* = 0.045), and specifically *O*. *anthropi* (R^2^ = 51.6%, *P* = 0.045). There were also significant correlations with biochemical pathways linked to glutamate and proline metabolism and separately with quorum sensing and biofilm formation which could be associated with monosaccharide production.

**Table 1:** Regression analysis for COPD patients using FEV_1_% of predicted, pack years and age.

	FEV_1_% of Predicted			Smoking Pack Years			Age		
	+ / -	R^2^ (%)	*P*	+ / -	R^2^ (%)	*P*	+ / -	R^2^ (%)	*P*
**Taxonomy**									
**Genus**									
***Streptococcus***	**+**	**51.8**	**0.044**	+	23.1	0.228	+	20.6	0.259
***Neisseria***	-	2.4	0.715	**-**	**66.1**	**0.014**	-	6.3	0.548
***Ochrobactrum***	-	13.4	0.373	-	3.1	0.679	**-**	**51.6**	**0.045**
**Species**									
***Streptococcus pneumonia***	**+**	**63.6**	**0.018**	+	8.4	0.487	+	40.1	0.092
***Ochrobactrum anthropi***	-	13.4	0.373	-	3.1	0.679	**-**	**51.6**	**0.045**
**Function**									
**Level 2**									
**Di- and oligosaccharides**	**+**	**50.8**	**0.047**	+	1.8	0.752	+	29.7	0.162
**Glutamine, glutamate, aspartate**	-	47.5	0.059	-	8.2	0.491	**-**	**78.7**	**0.003**
**Monosaccharides**	+	39.2	0.097	**+**	**3.8**	**0.000**	**+**	**55.2**	**0.035**
**Quorum sensing and biofilm form**	-	19.2	0.278	-	0.2	0.912	**-**	**52.9**	**0.041**
**Level 3**									
**Sialic Acid Metabolism**	**+**	**51.1**	**0.046**	+	18.1	0.293	+	40.0	0.092
**DNA repair, bacterial**	-	2.4	0.714	**-**	**74.8**	**0.006**	-	6.5	0.543
**Potassium homeostasis**	-	15.0	0.344	**-**	**65.6**	**0.015**	-	21.1	0.252
**YbbK**	-	3.4	0.662	**-**	**61.0**	**0.022**	-	18.0	0.294
**Glutamine, Glutamate, Aspartate and Asparagine Biosynthesis**	-	30.3	0.157	-	10.3	0.439	**-**	**66.5**	**0.014**
**Proline, 4-hydroxyproline uptake**	-	17.1	0.309	-	15.4	0.335	**-**	**53.3**	**0.040**

Regression analysis of FEV_1_% of predicted, commonly used as a measure of COPD severity, with normalised sequence numbers, reveals that the *Streptococcus* genus, and specifically *S*. *pneumonia* is positively correlated with FEV_1_% of predicted suggesting that it could act as a biomarker for COPD disease progression. Only those taxonomic or functional classifications found in all eight COPD patients, and with an R^2^ value of >0.5 were used in regression analysis. + or − symbols indicate whether relationship is positive or negative respectively. Significant regressions (*P* indicates significance *P* value of regression analysis) are highlighted in bold.

**Fig 5:**
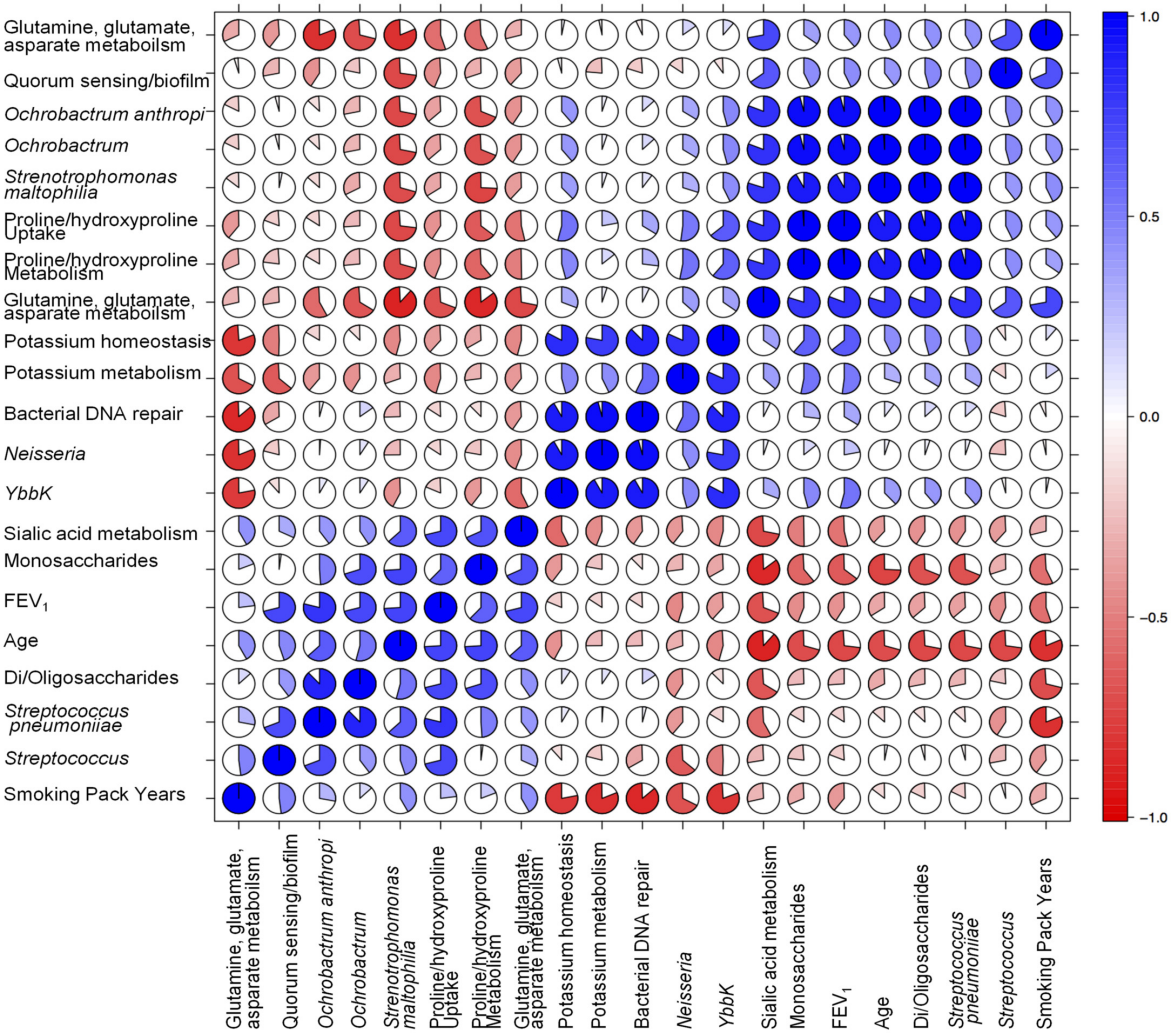
Multivariate comparisons of metagenomic variables displaying correlation coefficients. The pairwise correlation of multivariate parameters was performed by multiple Pearson analyses using the well-established correlograms (corrgrams) programme in R. The outputs are hierarchically clustered based on dissimilarity measures. The outputs are given in piecharts where the filled portion of the pie indicates the magnitude of the correlation and the depth of the shading indicates the magnitude of the correlation.

To expand our analyses, we conducted a pairwise multivariate correlations of variables (Fig 5). The correlation coefficient by corrgrams indicates a positive correlation (blue colour) and negative correlation (red colour) with the colour intensity indicative of the strength of the correlation. The order of variables is determined by the hierarchical clustering in which the correlation is the dissimilarity measure. R^2^ values are displayed in S3 Fig. Focusing on FEV_1_% of predicted, corrgrams analyses indicated positive correlations with mono, di and oligo-saccharides, age, and sialic acid metabolism indicative of sugar changes within the sputum linked to COPD severity. FEV_1_% of predicted significantly correlated with changes in the *Streptococcus* class / *Streptococcus pneumoniae* but with no other bacterial species. Age may have been a confounding factor in our analysis of the COPD metagenome, and in fact, age appeared to exhibit distinctive correlations with a number of metagenome features. Significant negative correlations with age were seen with proline, hydroxyproline, glutamine and glutamate metabolism and uptake indicative of altered, most likely reduced, bacterial nitrogen (N) metabolism as the patients aged. Negative correlations with age were seen with *Ochrobacterium* sp. and *Stenotrophomonas maltophilia*. The abundance of these species appeared to be strongly correlated with alterations in proline, hydroxyproline, glutamate and glutamine metabolism. Examining other strong correlations reveals a particularly strong node positively correlates *Neisseria* sp., *YbbK* and bacterial DNA repair. These appear to be negatively correlated with smoking pack years but not with FEV_1_% of predicted. Smoking pack years also negatively correlated with potassium homeostasis and metabolism.

## Discussion

The role of microbial pathogens in COPD has been well documented, specifically in relation to exacerbations [23]. These have included studies of scale microbial (“microbiomic”) changes as COPD progresses [4–6]. However, due to the limitations of the extent of sequence information obtained from (for example) 16S rRNA amplicons these have not unambiguously identified the species present. Further, functions of the bacterial population can at best only be suggested. Our study therefore, being the first metagenomic study of the microbiome in patients with COPD provides a more accurate description of the bacterial population structure down to the species level of resolution. Further, by providing wide-ranging bacterial genomic information we provide more robust descriptions of how bacterial changes impact on changes in gene function within the context of the whole microbiomic population.

The clinical potential offered by metagenomics approaches has been recently highlighted by a study of the human gut [24]. One of the suggestions arising from this work was that changes in certain functional classes could be used as a personalised disease risk factor. This is a valid possibility given increasingly reducing sequencing costs and the accessibility of DNA sequencing platforms. Within the context of COPD, any personalised medicine strategy would need to be based on minimally invasive sampling, thus, our study was based on spontaneous sputum rather than BAL sampling [25]. A wide range of features have been suggested as biomarkers for the progression of COPD making risk stratification of patient cohorts to improve monitoring and treatment plausible [26]. The microbiome may prove to be an effective source of such biomarkers in COPD and has already been suggested as useful for risk stratification in idiopathic pulmonary fibrosis [27].

COPD is a highly heterogeneous disease but, despite considerable variation between individual samples, we found significant differences between the COPD and ‘healthy’ smokers control groups. These are likely to reflect shifts in the species make up with the UBT microbiome so that they become sufficiently prominent to be detected using our sequencing technology. Importantly, we noted increases in four bacterial species—all pathogens—to above detection limits, only in COPD patients. Interestingly, although none of our patients were exacerbating, we commonly found *S*. *aureus* and *S*. *maltophilia* in the UBT microbiome of COPD patients although these have been linked with acute exacerbation [28]. Indeed, *S*. *maltophilia* has also been linked to exacerbations in cystic fibrosis patients [29]. Our observations, could suggest that these species may also be linked to reduced FEV_1_% of predicted as much as exacerbation. Thus, besides offering increased understanding of the developing underlying pathology, these four bacterial species could act as biomarkers for higher risk COPD patients.

Some reports have indicated that patients with severe COPD had a high prevalence of *P*. *aeruginosa*, *H*. *influenzae* and *S*. *pneumoniae* [30,31]. Our analyses did not suggest any significant correlation between *P*. *aeruginosa* and FEV_1_% of predicted. However, as *P*. *aeruginosa* does not appear to be part of the detected core UBT microbiome of our baseline COPD patients, it may be that any change in the abundance of this opportunistic pathogen is linked to exacerbation rather than COPD severity [32]. Conversely, *H*. *influenzae* was part of the common and possibly ‘core’ microbiome and did not change in abundance in our COPD patients. Taken together, these observations indicate that abundance changes in these bacteria species would be poor biomarkers for COPD progression. We did find a significant positive correlation with *S*. *pneumonia* and FEV_1_% of predicted, suggesting that as airflow obstruction increases (i.e. low FEV_1_% of predicted), the percentage abundance of *S*. *pneumonia* decreases. *S*. *pneumonia* is frequently cultured from the sputum samples of patients during exacerbations [30,31], and it is the main target of initial treatment with penicillin antibiotics. Detecting subtle changes *S*. *pneumonia* load, may allow prediction of COPD progression and allow earlier interventions.

In considering how patient drug history could be biasing our analyses were noted that of our eight COPD participants, six were currently prescribed inhaled corticosteroids. Some studies into the COPD microbiome have suggested that such treatments have an effect on its taxonomic composition [4,9], these samples did not appear to be outliers in (for example) our PCA studies, although the number of COPD patients not on inhaled corticosteroids did not allow statistically valid analyses to be performed.

Metagenomic analyses focusing on gene function indicated that there were increases in the abundance of functional alignments associated with bacterial growth, particularly bacterial cell division, nucleosides and nucleotides, and amino acid, carbohydrate, DNA, protein and RNA metabolism. These observations suggesting increased bacterial cell division were in line with a non-microbiome study where greater bacterial load has been linked to periods of COPD exacerbation [9]. We also noted increases in genetic factors linked to horizontal gene transfer which could indicate that large-scale genetic exchanges may be a characteristic of the COPD UBT microbiome, similar to the bacterial genomic flux which appears to be a feature of cystic fibrosis patients [33]. Our functional analysis also revealed the significant increase in alignments to the heat shock dnaK gene cluster, which in bacteria is responsible for producing the heat shock protein Hsp70. Analogues of Hsp70 have been shown to have significant anti-inflammatory responses in many inflammatory diseases [34]. Thus, Hsp70 could provide a mechanism for bacterial defence from the inflammatory mediators inherent within the lungs of COPD patients.

A further, significant, positive correlation was observed between FEV_1_% of predicted and the percentage abundance of genes associated with sialic acid metabolism; i.e. they decreased as COPD symptoms worsened. Sialic acids are nine carbon sugars backbone monosaccharides mainly decorating the outside of vertebrate cells, but also some microbes [35–37]. Extracellular sialic acid moieties have many roles in vertebrate immunology and can act to mask cell surface receptors or act as recognition sites for various lectins and antibodies. These roles include the modulation of leukocyte trafficking via selectins and influencing complement activation [38]. Sialic acid binding immunoglobulins (Ig)-like lectins (siglecs) are found in immune cells and will recognise different linkage-specific sialic acids. Examples of siglecs are siglec-3/CD33 related-siglecs found on haematopoietic cell lineages, siglec-9 on natural killer (NK) cells and siglec-8 only on circulating eosinophils. After binding sialyated moieties, siglecs can drive the internalisation of sialyated pathogens and crucially, modulate pathogen-/damage-associated molecular patterns (PAMP/DAMP)-mediated inflammation along with inhibition of NK cell activation. Sialic acid-Siglec interaction therefore, serves to maintain a baseline non-activated state of innate immune cells, and limit inflammatory response activation through PAMP/DAMP recognition [39]. The pathological advantages to the pathogen of acquired sialic acid decoration is therefore to augment siglec mediated avoidance of PAMP/DAMP recognition [39–41]. Additionally, the presence of sialylated lipopolysaccharide on the bacterial surface can prevent complement activation by binding to the C3 component of the complement cascade [42]. Our study suggests that with decreasing FEV_1_% of predicted scores decreasing sialic metabolite would indicate a shift towards a lesser capacity to avoid recognition and thus suppress inflammation—a key feature of COPD. Thus, a reduction in sialic acid metabolising capacity in the bacteria bacterial population could be an important pathological feature in COPD progression.

To substantiate the observed significant correlations with FEV_1_% of predicted, we noted no significant relationships with either smoking pack years, or the age of COPD patients. However, although our patient cohort is relatively large for metagenomic sequencing studies, its size does mean that interpretation of regression significance values should be taken with care, particularly with regards to those close to our significance threshold, *P* < 0.05, such as those below 0.1. Here, we have associated a number of taxonomic and functional features of the microbiome with COPD severity. Of these, two features, *S*. *pneumonia* and sialic acid metabolism have regression associations with the age of COPD patients with significance *P* values of less than 0.1. Albeit not significant, these values suggest that in a longitudinal study associating COPD severity and progression with taxonomic and functional features of the microbiome, the age of COPD patients may be a confounding variable that needs to be controlled for.

Within this study, we indirectly identified significant relationships between smoking and the *Neisseria* genus, which has previously been linked to smoking [43], and a number of Level 3 functional classifications, namely bacterial DNA repair and potassium homeostasis. These features possibly reflect, smoking linked bacterial genomic damage and a response to the inclusion of potassium salts in cigarette papers, respectively [44,45].

It should be noted that here we have used metagenomic sequencing of genomic DNA to assess the functional capacity of the UBT microbiome in COPD patients. This gives an accurate profile of the genetic capacity of the microbiome and the possible selective pressures acting upon it. However, it is not able to provide information on the genetic expression of the COPD microbiome. To allow for this, metatranscriptomic sequencing of RNA from the COPD microbiome would be required. This could be used to determine how expression of the functional capacity of the COPD microbiome relates to clinical parameters, as has been shown in other respiratory conditions [46].

Although based on a relatively small number of patient samples; we have shown the potential of metagenomic sequencing to give novel insights into COPD. In addition to identifying potential novel bacterial and functional biomarkers for COPD progression, it has also demonstrated the potential strengths of using metagenomic techniques to characterise the COPD microbiome from an easily accessible biofluid in patients with COPD. Future studies should report metagenomic profiles to hospital admissions, rate of FEV_1_% of predicted decline, and mortality in larger numbers as, ultimately, it raises potential avenues for improving, and even personalising, diagnostics and treatment regiments.

## Supporting Information

S1 Fig Significant changes in Level 1 functional abundance from Control to COPD. Using MetaboAnalyst 2.0, t-Tests and fold-changes were calculated from normalised percentages of reads, with only those with a *P* value of < 0.05 charted. Of the four Level 1 functional classifications, three are increased in COPD samples, and one is decreased.
(DOCX)

S2 Fig Significant changes in Level 2 functional abundance from Control to COPD. Using MetaboAnalyst 2.0, t-Tests and fold-changes were calculated from normalised percentages of reads, with only those with a *P* value of < 0.05 charted. Of the 26 Level 2 functional classifications that are significantly different, only four are decreased in COPD, suggesting a greater degree of selective pressure in the upper respiratory tract of COPD patients.
(DOCX)

S3 Fig Significant correlation coefficients of the metagenomic variables as defined using corrgrams. Using the corragrams programme pairwise correlation of the metagenomic variables were derived. The order of variables reflects the hierarchical clustering in which the correlation is the dissimilarity measure. The top half of the correlation matrix reflects the R^2^ values with the font size reflecting the degree of significance. The lower half plots the individual pairwise correlations and regression lines.
(DOCX)

S1 Table Individual participant details, and clinical information for COPD patients. Full participant information for Control participants and COPD patients, showing age, gender, and smoking history. Additional clinical information for COPD patients includes drug history, medical history, FEV_1_% of predicted, and whether the patient had an infection at the time of giving a sample. nc = not collected.
(DOCX)

S2 Table Sequencing Statistics for Control and COPD groups. Average read statistics pre and post quality control (QC), after merging of paired-end reads, alongside corresponding one-way ANOVA *P* values. Analysis shows no significant differences in all but one read characteristic, average read length both pre and post QC, suggesting that the HiSeq 2500 sequencing approach and MG-RAST analysis pipeline introduced no discernible bias between the two participant groups.
(DOCX)
