# Supplementary material for: Metagenomic Sequencing of the Chronic Obstructive Pulmonary Disease Upper Bronchial Tract Microbiome Reveals Functional Changes Associated with Disease Severity
Source: PLoS One. 2016 Feb 12;11(2):e0149095. doi: 10.1371/journal.pone.0149095 (PMC4752236; doi:10.1371/journal.pone.0149095)
Supplement: S2 Fig — Using MetaboAnalyst 2.0, t-Tests and fold-changes were calculated from normalised percentages of reads, with only those with a P value of < 0.05 charted. Of the 26 Level 2 functional classifications that are significantly different, only four are decreased in COPD, suggesting a greater degree of selective pressure in the upper respiratory tract of COPD patients. (DOCX) [file pone.0149095.s002.docx]

**Sup. Fig. 2**
