# Supplementary figures and images for: Metagenomic Sequencing of the Chronic Obstructive Pulmonary Disease Upper Bronchial Tract Microbiome Reveals Functional Changes Associated with Disease Severity
Source: PLoS One. 2016 Feb 12;11(2):e0149095. doi: 10.1371/journal.pone.0149095 (PMC4752236; doi:10.1371/journal.pone.0149095)

**Sup. Fig. 3**


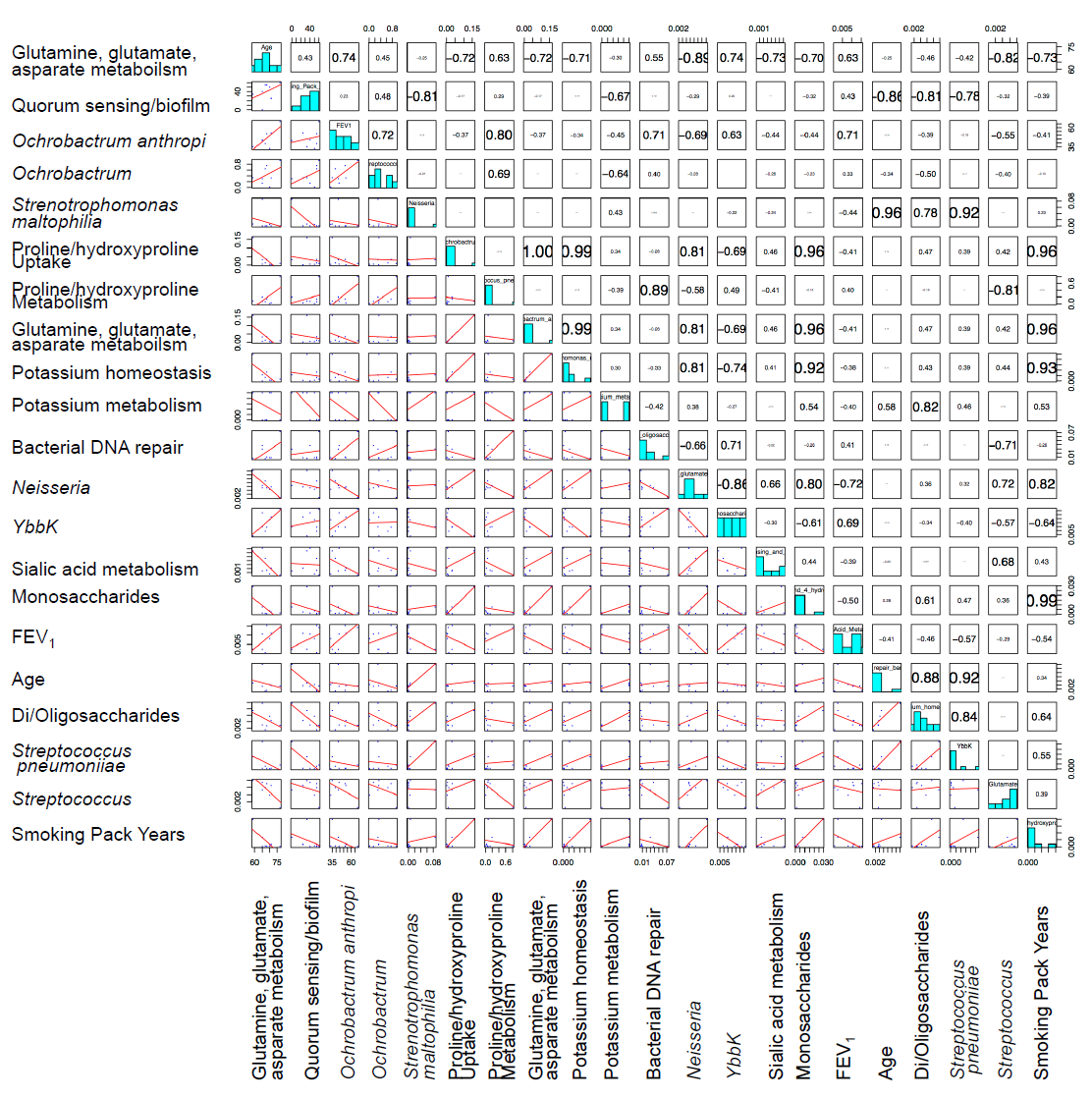

Supplement: S3 Fig — Using the corragrams programme pairwise correlation of the metagenomic variables were derived. The order of variables reflects the hierarchical clustering in which the correlation is the dissimilarity measure. The top half of the correlation matrix reflects the R2 values with the font size reflecting the degree of significance. The lower half plots the individual pairwise correlations and regression lines. (DOCX) [file pone.0149095.s003.docx]
