# Supplementary material for: Metagenomic Sequencing of the Chronic Obstructive Pulmonary Disease Upper Bronchial Tract Microbiome Reveals Functional Changes Associated with Disease Severity
Source: PLoS One. 2016 Feb 12;11(2):e0149095. doi: 10.1371/journal.pone.0149095 (PMC4752236; doi:10.1371/journal.pone.0149095)
Supplement: S1 Table — Full participant information for Control participants and COPD patients, showing age, gender, and smoking history. Additional clinical information for COPD patients includes drug history, medical history, FEV1% of predicted, and whether the patient had an infection at the time of giving a sample. nc = not collected. (DOCX) [file pone.0149095.s004.docx]

**Sup. Table 1**

| **Sample ID** | **MG-RAST ID and Link** | **Age** | **Sex** | **Drug History** | **Medical History** | **Smoking Status** | **Smoking Pack Years** | **Patient Infection** | **FEV_1_** | **GOLD Rating** |
| --- | --- | --- | --- | --- | --- | --- | --- | --- | --- | --- |
| SCON01 | [4549011.3](http://metagenomics.anl.gov/linkin.cgi?metagenome=4549011.3) | 54 | F | *nc* | *nc* | Ex | *nc* | *nc* | *nc* | *nc* |
| SCON02 | [4552904.3](http://metagenomics.anl.gov/linkin.cgi?metagenome=4552904.3) | 43 | M | *nc* | *nc* | Ex | *nc* | *nc* | *nc* | *nc* |
| SCON03 | [4552905.3](http://metagenomics.anl.gov/linkin.cgi?metagenome=4552905.3) | 81 | M | *nc* | *nc* | Ex | *nc* | *nc* | *nc* | *nc* |
| SCON04 | [4549020.3](http://metagenomics.anl.gov/linkin.cgi?metagenome=4549020.3) | 62 | M | *nc* | *nc* | Current | *nc* | *nc* | *nc* | *nc* |
| SCON05 | [4549017.3](http://metagenomics.anl.gov/linkin.cgi?metagenome=4549017.3) | 56 | F | *nc* | *nc* | Current | *nc* | *nc* | *nc* | *nc* |
| SCON06 | [4552983.3](http://metagenomics.anl.gov/linkin.cgi?metagenome=4552983.3) | 58 | M | *nc* | *nc* | Ex | *nc* | *nc* | *nc* | *nc* |
| SCON07 | [4552906.3](http://metagenomics.anl.gov/linkin.cgi?metagenome=4552906.3) | 56 | F | *nc* | *nc* | Current | *nc* | *nc* | *nc* | *nc* |
| SCON08 | [4549021.3](http://metagenomics.anl.gov/linkin.cgi?metagenome=4549021.3) | 40 | M | *nc* | *nc* | Ex | *nc* | *nc* | *nc* | *nc* |
| SCON09 | [4549018.3](http://metagenomics.anl.gov/linkin.cgi?metagenome=4549018.3) | 44 | M | *nc* | *nc* | Ex | *nc* | *nc* | *nc* | *nc* |
| SCON10 | [4549014.3](http://metagenomics.anl.gov/linkin.cgi?metagenome=4549014.3) | 35 | F | *nc* | *nc* | Current | *nc* | *nc* | *nc* | *nc* |
| COPD01 | [4548729.3](http://metagenomics.anl.gov/linkin.cgi?metagenome=4548729.3) | 77 | F | Carbocysteine,Simvastatin,Prednisolone,Salbutomol, Fluticason.Paracetomol | Lower lobe collapse | Current | 60 | No | 68 | II |
| COPD02 | [4548728.3](http://metagenomics.anl.gov/linkin.cgi?metagenome=4548728.3) | 67 | F | Dihydrocodein,Seretide,Tiotropium,Mirapexin,Amitriptyline | Parkinsons | Current | 55 | No | 39 | III |
| COPD03 | [4548727.3](http://metagenomics.anl.gov/linkin.cgi?metagenome=4548727.3) | 65 | M | Prednisolone,Carbocisteine,Symbicort,Spiriva,Salbutomol, Alendronic acid,Arithromyocin | Asthma, Prostate, Osteoporosis, Cataract, | Never | 0 | No | 43 | III |
| COPD05 | [4549506.3](http://metagenomics.anl.gov/linkin.cgi?metagenome=4549506.3) | 65 | M | Carbocystine, Prednisolone, Salbutomol, Seretide, Tiotropium, Simvastatin, Lansoprazole | Kidney disease, Pneumonia, Vocal cord paralysis | Current | 40 | No | 44 | III |
| COPD06 | [4549855.3](http://metagenomics.anl.gov/linkin.cgi?metagenome=4549855.3) | 70 | M | Metformin, Gabapentin, Glicazide, Nifedipine, Frusemide, Simvastatin, Tramadol, Alfuzosin, Ipratropium bromide | TIA, Type 2 diabetes, Spinal cord stenosis, Obesity | Ex | 50 | No | 33 | III |
| COPD07 | [4552977.3](http://metagenomics.anl.gov/linkin.cgi?metagenome=4552977.3) | 68 | M | Zithromax, Valsartin, Atenolol, Metformin, Gliclazide, Carbocisteine, Symbicourt, Spiriva, Ventolin, GTN | Bilary Sepsis, Bronchiectasis | Current | 55 | No | 52 | II |
| COPD04 | [4548730.3](http://metagenomics.anl.gov/linkin.cgi?metagenome=4548730.3) | 59 | M | Fluoxetine, Seretide, Salbutomol, Tiotropium | None | Ex | 34 | No | 35 | III |
| COPD08 | [4552978.3](http://metagenomics.anl.gov/linkin.cgi?metagenome=4552978.3) | 71 | F | mirtazipine; prochlorperizine; symbicort; amlodipine; bendrofluazide | COPD; TB; hypertension | Current | 25 | No | 53 | II |
