# Supplementary material for: Metagenomic Sequencing of the Chronic Obstructive Pulmonary Disease Upper Bronchial Tract Microbiome Reveals Functional Changes Associated with Disease Severity
Source: PLoS One. 2016 Feb 12;11(2):e0149095. doi: 10.1371/journal.pone.0149095 (PMC4752236; doi:10.1371/journal.pone.0149095)
Supplement: S2 Table — Average read statistics pre and post quality control (QC), after merging of paired-end reads, alongside corresponding one-way ANOVA P values. Analysis shows no significant differences in all but one read characteristic, average read length both pre and post QC, suggesting that the HiSeq 2500 sequencing approach and MG-RAST analysis pipeline introduced no discernible bias between the two participant groups. (DOCX) [file pone.0149095.s005.docx]

**Sup. Table 2**

|  | **COPD** | | **Control** | | ***P* Value** |
| --- | --- | --- | --- | --- | --- |
|  | **Average** | **St. Dev** | **Average** | **St. Dev** |  |
| **Pre QC bp Count** | 4.53 x 10^9^ | 2.23 x 10^9^ | 2.56 x 10^9^ | 5.88 x 10^8^ | 0.195 |
| **Pre QC Sequence Count** | 2.87 x 10^7^ | 1.43 x 10^7^ | 1.63 x 10^7^ | 2.93 x 10^6^ | 0.194 |
| **Pre QC Length (bp)** | 158.00 | 1.00 | 163.00 | 1.34 | **0.001** |
| **Pre QC Mean GC %** | 42.40 | 0.26 | 42.20 | 0.61 | 0.712 |
| **Pre QC Artificial Duplicate Reads** | 1.60 x 10^6^ | 1.02 x 10^6^ | 8.80 x 10^5^ | 3.99 x 10^5^ | 0.319 |
| **Post QC bp Count** | 2.35 x 10^9^ | 1.23 x 10^9^ | 1.20 x 10^9^ | 3.20 x 10^8^ | 0.171 |
| **Post QC Sequence Count** | 1.80 x 10^7^ | 9.54 x 10^6^ | 8.48 x 10^6^ | 2.10 x 10^6^ | 0.144 |
| **Post QC Length (bp)** | 132.00 | 1.39 | 140.00 | 2.41 | **0.001** |
| **Post QC Mean GC %** | 42.80 | 0.23 | 42.90 | 0.44 | 0.668 |
| **Predicted Protein Features** | 1.37 x 10^7^ | 7.14 x 10^6^ | 6.75 x 10^6^ | 1.88 x 10^6^ | 0.158 |
| **Predicted rRNA Features** | 5.21 x 10^6^ | 2.59 x 10^6^ | 2.86 x 10^6^ | 4.49 x 10^5^ | 0.175 |
| **Identified Protein Features** | 8.69 x 10^5^ | 3.88 x 10^5^ | 2.11 x 10^6^ | 1.54 x 10^6^ | 0.283 |
| **Identified rRNA Features** | 2.09 x 10^4^ | 9.75 x 10^3^ | 3.35 x 10^4^ | 2.34 x 10^4^ | 0.488 |
| **Identified Functional Categories** | 1.63 x 10^5^ | 7.35 x 10^4^ | 1.02 x 10^6^ | 8.66 x 10^5^ | 0.185 |
| **Archaea** | 107.00 | 31.83 | 3.84 x 10^3^ | 4.17 x 10^3^ | 0.227 |
| **Bacteria** | 2.21 x 10^5^ | 7.44 x 10^4^ | 3.49 x 10^6^ | 3.16 x 10^6^ | 0.166 |
| **Eukaryota** | 9.80 x 10^5^ | 4.51 x 10^5^ | 4.18 x 10^5^ | 9.69 x 10^4^ | 0.071 |
| **Viruses** | 4.51 x 10^3^ | 2.07 x 10^3^ | 6.13 x 10^3^ | 3.97 x 10^3^ | 0.611 |
| **Other** | 2.36 x 10^4^ | 1.35 x 10^4^ | 7.08 x 10^3^ | 2.81 x 10^3^ | 0.074 |
| **Total Taxonomic Count** | 1.23 x 10^6^ | 5.31 x 10^5^ | 3.92 x 10^6^ | 3.10 x 10^6^ | 0.245 |
